# Two cases about mesh adhesion to intra-abdominal cavity tissue after using mesh to repair an incisional hernia

**DOI:** 10.12669/pjms.334.12641

**Published:** 2017

**Authors:** Xuefeng Xia, Xiaofeng Lu, Xing Kang, Ji Miao, Kai Zhang, Wenxian Guan

**Affiliations:** 1 Xuefeng Xia, Department of General Surgery, The Affiliated Drum Tower Hospital of Nanjing University Medical School, Nanjing 210008, Jiangsu Province, China; 2 Xiaofeng Lu, Department of General Surgery, The Affiliated Drum Tower Hospital of Nanjing University Medical School, Nanjing 210008, Jiangsu Province, China; 3 Xing Kang, Department of General Surgery, The Affiliated Drum Tower Hospital of Nanjing University Medical School, Nanjing 210008, Jiangsu Province, China; 4 Ji Miao, Department of General Surgery, The Affiliated Drum Tower Hospital of Nanjing University Medical School, Nanjing 210008, Jiangsu Province, China; 5 Kai Zhang, Department of General Surgery, The Affiliated Drum Tower Hospital of Nanjing University Medical School, Nanjing 210008, Jiangsu Province, China; 6 Wenxian Guan, Department of General Surgery, The Affiliated Drum Tower Hospital of Nanjing University Medical School, Nanjing 210008, Jiangsu Province, China

**Keywords:** Incisional hernia, Mesh, Adhesion, Laparoscope

## Abstract

Abdominal incisional hernia is a common postoperative complication. With the development of a new type of surgical anti-adhesion mesh, mesh repair has become a widely-adopted procedure, particularly in the laparoscopic era. However, there were few reports about use of these new meshes to repair incisional hernia in the abdominal cavity. In this report, we present two cases: one a 72-year-old male and the other a 62-year-old female. Both of those patients suffered incisional hernias during abdominal operations, and therefore underwent open incisional hernia anti-adhesion mesh repair operations. Both of them had recurrent incisional hernias after the first repair operation. During the second hernia repair operation via laparoscopy, tissue from the intestine and omentum were found to have adhered seriously to the old meshes, which could cause many serious problems. We need to pay more attention to the issue of adhesion, try to determine possible reasons and improve in our future work.

## INTRODUCTION

Incisional hernia is a common postoperative complication which usually requires surgical repair.^1^ Ventral hernia repair is one of the most common surgical procedures, but the exact nature of the surgery depends of the surgeon’s preference and experience. Incisional hernia repair can be divided into suture repair and mesh repair and also open ventral hernia repair (OVHR) and laparoscopic ventral hernia repair (LVHR).^2^

Since Leblanc and Booth first described the application of the laparoscopic technique to hernia repair^3^, an increasing number of surgeons use LVHR since it is equally as effective as OVHR but carries a lower risk of complication and ventral hernia recurrence.^4^ Advances in anti-adhesion mesh laparoscopy have meant that most incisional hernias are repaired with an intraperitoneal mesh,^5^ which can remarkably reduce recurrence compared to suture repair. Most surgical anti-adhesion mesh used clinically is coated with substances including silicon; a hydrophilic membrane layer composed of collagen and polyethylene glycol; and expanded polytetrafluoroethylene. Besides the lower hernia recurrence rate, using anti-adhesion meshes can also reduce the occurrence of many other postoperative complications, such as infection, inflammation and pain, which can affect quality of life.^5^-^7^

However, there are few reports about the condition of the abdominal cavity after using these new types of mesh to repair incisional hernia. Here, we report two typical cases in our clinic work.

## CASE 1

**Fig. 1:**
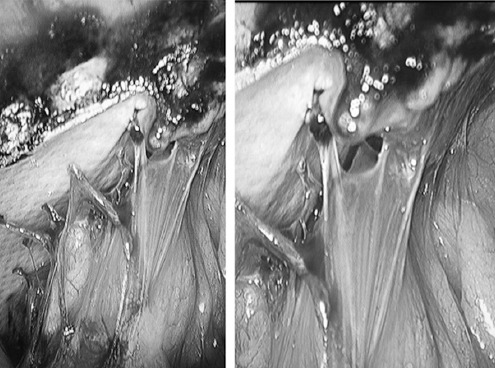
In case 1, we found severe adhesions of the intestinal tract and omentum to the mesh.

**Fig. 2:**
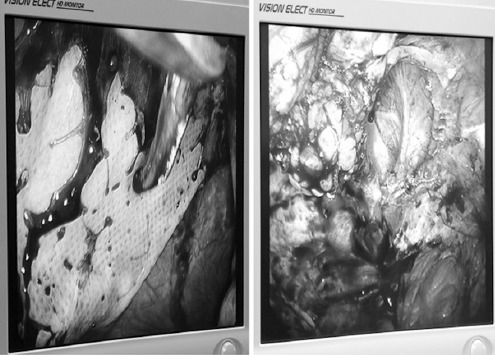
In case 1, the old mesh was intact and the location of the incisional hernia was the same as the original incision.

The patient was a 72-year-old man, who underwent open cholecystectomy in 1992 due to gallstones. After the first operation, a mass was found in the operative area which was diagnosed as an incisional hernia. This patient underwent open incisional hernia repair in 2015 using a TintraS mesh from Aspide Medical. A year later, a further mass was found in the same operative area which was diagnosed as a recurrent incisional hernia. Therefore, we decided to repair the hernia laparoscopically. During the operation, we found severe adhesions of the intestinal tract and omentum to the mesh used during the previous incisional hernia repair (Fig. 1). After separating the adhesion, we found that the old mesh was intact and the location of the incisional hernia was the same as the original incision (Fig. 2). A 3-0 Prolene suture was used to close the abdominal wall defect and a larger anti-adhesion mesh was placed to cover the defect. This patient recovered well and was discharged four days post-operatively without any major discomfort. Follow-up observation showed a good result.

## CASE 2

**Fig. 3:**
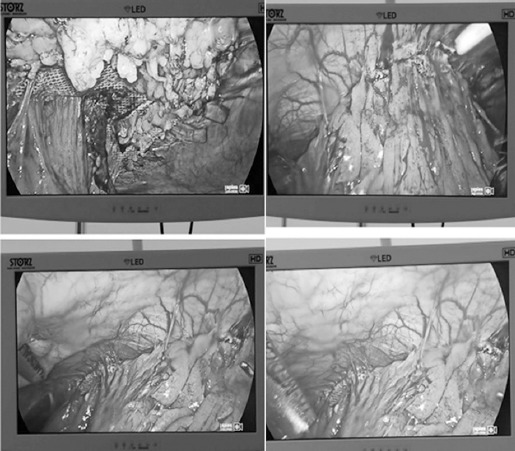
In case 2, we also found severe adhesions of the intestinal tract and omentum to the mesh.

The patient was a 62-year-old woman who underwent surgery due to endometrial cancer in 2002. After the first operation, she was diagnosed with an incisional hernia and underwent open incisional hernia repair in 2012 using a Parietex™ Composite mesh. A mass was found in the same area three years later and was diagnosed as a recurrent incisional hernia. We therefore used laparoscopy to repair the hernia. During the operation, we found severe adhesions of the omentum, mesentery and intestinal tract to the mesh (Fig. 3). After separating the adhesion, the old mesh was also found to be intact and the site of the incisional hernia was the same as the original incision. We used a 3-0 Prolene suture to close the defect and placed a larger anti-adhesion mesh to cover the defect. This patient recovered well and was also discharged four days post-operatively Follow-up observation also showed a good result.

## DISCUSSION

Since the 1950s, when tantalum meshes were first used to repair ventral hernias,^14^,^15^ various meshes have been used in clinical practice.^3^,^8^-^13^ Mesh repair helps to reduce the high hernia recurrence rate from over 50% to 20%.^16^ Use of a mesh to reinforce the abdominal wall is considered the gold standard in OVHR,^16^ since the mesh strengthens the abdominal wall defect without tension.

LVHR is now widely accepted for the repair of ventral hernia defects.^17^ It has the advantages of a shorter hospital stay, lower incidence of infection, less pain and less risk of adhesion.^17^,^18^ There are some common complications following LVHR, including seroma formation, ileus, pain and wound infection.^19^ More serious complications include bowel injury, which is higher in LVHR than OVHR.^18^,^20^ Although lower than in OVHR, the hernia recurrence rate after LVHR has been reported to be up to 9%.^19^

Since they are allogeneic, a prosthetic could lead to inflammation resulting in permanent adhesions between the mesh and abdominal viscera,^21^,^22^ especially if it is located intraperitoneally. Adhesion may cause serious complications including chronic pain, intestinal obstruction and enterocutanic fistulae.^22^-^24^ Traditional meshes are made of propylene: whilst this is strong and stable, it easily causes adhesion.^22^,^25^,^26^ When adhesion occurs, it is recommended to implant an anti-adhesion mesh, a novel prosthesis coated in a material to lower adhesion. Using an anti-adhesion mesh for repair could reduce the risk of recurrence, infection, inflammation and pain, which can improve patients’ post-operative life quality.^5^-^7^

The similarity in these two cases is that both patients suffered abdominal operations more than once, and also both protopathic and previous incision hernia repair operations were open surgery and used anti-adhesion meshes. To repair the recurrent incisional hernias, we used laparoscopy in both cases. We found serious adhesions of the omentum, mesentery and intestinal tract to the old mesh. Since there are few reports about these kinds of cases, we put forward probable causes.

### 1. The Mesh

Almost all types of surgical anti-adhesion meshes have an anti-adhesion coating. However, these coating materials can only reduce the incidence rate or/and severity of adhesion and cannot entirely prevent post-operation adhesion since they are still considered foreign matter by the body. The mesh can stimulate local inflammation, even though the rate of serious adhesion is lower than with traditional materials, leading to recurrence or serious complications such as ileus. Sometimes, second operations are open, so there is no clear picture about mesh adhesions during these operations.

### 2. Operation technique

It is possible that the omentum or intestinal tissue was injured by the operation technique during the previous surgery, which could lead to inflammation, effusion or other protopathy. In turn, this could cause adhesion to the parietal peritoneum. When separating the adhesion, it is likely that the tissue was further damaged, again leading to inflammation and/or effusion, possibly leading to further adhesion. In addition, we found that defects from the incision during the first operation were still present and that the incision had not been closed during the second repair operation. We suggest that the adhesion could be related to this detail. Since both first repair operations were open, the incisions were larger than with LVHR, increasing the severity of inflammation, which could increase the risk of adhesion. We suggest that using LVHR might lower the incidence of adhesion after operation.

### 3. The patients

Incision hernia is a common complication after abdominal surgery, and many cases require a repair operation. However, there are few reports similar to the patients presented in this study, so we could not rule out the possibility that the above two cases are special and due to a specific aspect of the patients’ physical condition.

### 4. Failure of the first repair operation

Both patients underwent an open incision hernia repair operation, and we found both of the second hernias were recurrence incisional hernias. This indicated that the first repair was not successful, and this might be the main reason causing the recurrence and adhesion.

Due to improvements in surgical technique and the new types of mesh, recurrence and complication incidence rates are lower. In addition, it is difficult to observe the condition of the abdomen after mesh repair without a second operation, especially without a laparoscope. However, mesh adhesion is still a problem, as demonstrated by the two cases presented here. Adhesion can cause many other abdominal problems, such as ileus and chronic abdominal pain.^7^ Further research could identify the reasons for adhesions and inform future focus, for example whether we need to improve surgical skills, carry out repair by using laparoscopically or even improve the coating composition.
